# Parental COVID-19 vaccine hesitancy for children: vulnerability in an urban hotspot

**DOI:** 10.1186/s12889-021-11725-5

**Published:** 2021-09-13

**Authors:** Nina L. Alfieri, Jennifer D. Kusma, Nia Heard-Garris, Matthew M. Davis, Emily Golbeck, Leonardo Barrera, Michelle L. Macy

**Affiliations:** 1 Division of Advanced General Pediatrics and Primary Care, Ann & Robert H. Lurie Children’s Hospital of Chicago, 225 E Chicago Ave, Box 162, Chicago, IL 60611 USA; 2 Mary Ann & J. Milburn Smith Child Health Outcomes, Research, and Evaluation Center; Stanley Manne Children’s Research Institute, Ann & Robert H. Lurie Children’s Hospital of Chicago, Chicago, IL USA; 3 Department of Pediatrics, Northwestern University Feinberg School of Medicine, Chicago, IL USA; 4 Department of Medicine, Medical Social Sciences, and Preventive Medicine, Northwestern University Feinberg School of Medicine, Chicago, IL USA; 5 Division of Emergency Medicine, Ann & Robert H. Lurie Children’s Hospital of Chicago, Chicago, IL USA

**Keywords:** COVID-19, Vaccine hesitancy, Health equity

## Abstract

**Objective:**

To compare hesitancy toward a future COVID-19 vaccine for children of various sociodemographic groups in a major metropolitan area, and to understand how parents obtain information about COVID-19.

**Methods:**

Cross-sectional online survey of parents with children < 18 years old in Chicago and Cook County, Illinois, in June 2020. We used logistic regression to determine the odds of parental COVID-19 vaccine hesitancy (VH) for racial/ethnic and socioeconomic groups, controlling for sociodemographic factors and the sources where parents obtain information regarding COVID-19.

**Results:**

Surveys were received from 1702 parents and 1425 were included in analyses. Overall, 33% of parents reported VH for their child. COVID-19 VH was higher among non-Hispanic Black parents compared with non-Hispanic White parents (Odds Ratio (OR) 2.65, 95% Confidence Interval (CI): (1.99–3.53), parents of publicly insured children compared with privately insured (OR 1.93, (1.53–2.42)) and among lower income groups. Parents receive information about COVID-19 from a variety of sources, and those who report using family, internet and health care providers as information sources (compared to those who don’t use each respective source) had lower odds of COVID-19 VH for their children.

**Conclusions:**

The highest rates of hesitancy toward a future COVID-19 vaccine were found in demographic groups that have been the most severely affected by the pandemic. These groups may require targeted outreach efforts from trusted sources of information in order to promote equitable uptake of a future COVID-19 vaccine.

## Background

The COVID-19 pandemic has resulted in high levels of disease and death tolls [1, 2]. Of the over 175 million cases worldwide to date as of June 2021, 33.5 million have been in the United States [2] with Black and Hispanic communities experiencing an increased burden of hospitalizations and deaths compared to White counterparts. In the initial wave of the pandemic, urban centers were amongst the hardest-hit areas. Cook County, which includes the city of Chicago, has had over 270,000 cases and over 5000 deaths, as of June 2021 [3], with Black and Hispanic hospitalizations and deaths far surpassing the rates of non-Hispanic Whites respectively [3, 4]. To mitigate the effects and spread of the COVID-19 pandemic, several important public health measures have been found effective, including universal mask wearing, maintaining physical distances, and limiting social contact. In addition to these measures, two vaccines in the United States have received emergency authorization, with one approved for individuals as young as 12 years old as of June 2021. When the vaccine initially received emergency authorization, vaccines were being administered to priority groups such as health care professionals, essential workers, and individuals over the age of 65, with guidelines subsequently expanding [5]. Vaccine data from clinical trials has shown promise in preventing recipients from morbidity and mortality due to COVID-19, and there is hope for decreasing viral transmission and therefore disease volume and burden [5].

In order for a vaccine to be effective in controlling the spread of COVID-19 it is estimated that 67% of the population will need to receive the vaccine to reach herd immunity [6]. Recent data suggests that countries like the United States may never achieve herd immunity, but as children make up 22% of the American population, including children in vaccination efforts and plans is imperative for increasing community protection against COVID-19 [7, 8]. With vaccination efforts underway for some adults, it is important to recognize that many adults themselves may decline or defer the vaccine. Unfortunately, vaccine hesitancy, skepticism, and refusal have been a longstanding public health problem for routine immunizations and the annual influenza vaccine for both children and adults. Lower vaccine confidence and uptake has been observed in certain sociodemographic groups including racial/ethnic minorities [9, 10] and those with lower socioeconomic status [11].

Understanding the differences in COVID-19 vaccine hesitancy across varying communities and sociodemographic groups is critical for the identification of populations for whom the currently available COVID-19 vaccine information may be insufficient to promote uptake. With this knowledge, vaccine communication and distribution strategies could be tailored to hesitant groups. The objectives of this study were twofold: 1) to compare COVID-19 vaccine hesitancy in sociodemographic groups with higher rates of COVID-19 versus less affected groups, and, 2) to understand how members of these groups receive information about COVID-19 during the first wave of the pandemic.

## Methods

This study consisted of a cross-sectional online survey of parents with children < 18 years who live in the Chicago metropolitan area and the surrounding suburbs that make up Cook County, IL, as the area was beginning to reopen after the first wave of COVID-19 in June 2020.

### Setting and participants

We used the Qualtrics^XM^ online survey platform to distribute our survey to parents representative of the racial and ethnic composition of Cook County based on 2010 US Census data. This was done purposefully, using panels of existing survey participants.

### Survey instrument development

The study team developed unique survey questions and also incorporated items from previously published surveys regarding parents and children’s responses to epidemics, disasters such as hurricanes, and pandemics including Zika virus [12–15]. We pre-tested the survey with 27 respondents on the Qualtrics^XM^ platform to ensure proper survey functionality and clarity.

### Measures

#### Sociodemographic measures

Our survey assessed demographic characteristics including respondent race and ethnicity, family income, and child insurance type. Race and ethnicity information was collected by asking respondents “what is your race?” and “what is your ethnicity” as two distinct questions. From these responses, a composite race/ethnicity variable was created: non-Hispanic White, non-Hispanic Black, Hispanic, and non-Hispanic mixed-race/other. We asked respondents to estimate their annual household income in categories based on federal poverty levels including: “< $39,999”, “$40,000–$79,999”, “$80,000–$149,999”, “> $150,000”, “I’m not sure”, or “prefer not to answer”. We collapsed no answer, I’m not sure, and prefer not to answer into one category we labeled “did not disclose”. Insurance type was identified by asking if their child had private, public, or no insurance.

#### Sources of COVID-19 information measure

We asked respondents to select all that apply from an 11-item list to indicate how they received information about COVID-19. Response options included: family/friends/word of mouth, internet, social media, Department of Health/Centers for Disease Control (CDC)/Government Agency (“Government Agencies”), newspaper (print or online), TV, radio, podcasts, church/place of worship, health care provider, or other. In addition, we asked parents to indicate their level of confidence as “a lot”, “some”, or “little to no” in each information source they reported using. These two items (use of each source of information and confidence in each source of information) were combined into a new dichotomous variable that included “using” the source of information (having reported “a lot” or “some” confidence) versus “not using” the source (reporting “little to no” confidence or not using the source of information). This dichotomous variable was used in our logistic regression models.

#### COVID-19 vaccine hesitancy measure

To understand willingness to obtain a future COVID-19 vaccine, respondents were asked “If a new vaccine against COVID-19 became available, how likely would you be to get your child vaccinated?” Respondent parents answered for their child, with responses of “very likely”, “somewhat likely”, “not likely”, and “I’m not sure”. We dichotomized responses as having vaccine hesitancy (VH) for those who responded “not likely” or “not sure” and vaccine amenable for those who responded they were “very likely” or “somewhat likely” to get a future COVID-19 vaccine.

### Survey distribution

Recruitment occurred using Qualtrics^XM^ “dynamic surveys” and their online dashboard-style portal on which surveys are distributed to eligible panel members who log onto the portal and access surveys as they become available. The survey was made available in English and Spanish. Survey responses were collected from June 8, 2020 to June 29, 2020. To enhance response rates, Qualtrics^XM^ utilized an automated reminder option based on respondents’ preference.

To ensure data quality, the following checks were put into place [1] participants’ surveys were terminated if they were found to be “speeding” [16] as indicated by one-half the median of the completion speed during the pilot [2], only unique IP address were included to prevent duplicate responses, and [3] surveys were excluded if respondents chose the same answer for every question, also known as “straight lining” [16].

### Ethical considerations

The study was considered exempt human subjects research by the Ann & Robert H Lurie Children’s Hospital of Chicago Institutional Review Board. Incentives for participation were determined by Qualtrics^XM^ based on time spent completing the survey, and compensation on similar surveys, which Qualtrics^XM^ estimated to be about $5 per survey.

### Analysis

Our dichotomous outcome of interest was vaccine hesitancy (VH). Predictors of interest were race/ethnicity, income, insurance type, and sources of information. First, bivariate associations between VH and our predictors of interest were tested. Next we built a multivariate logistic regression model of COVID-19 VH, controlling for parent race/ethnicity, annual household income, child insurance type, and sources of information in which parents reported confidence. All covariates that were significant (*p* < 0.05) in the multiple variable model were retained. The sources of information that did not retain significance were dropped from the final model. A goodness-of-fit test for the adjusted logistic regression model was performed using the Hosmer-Lemeshow test, and was significant with a *p* value of 0.09.

Factor analysis and Cronbach alpha testing were performed to determine if we could group the sources of information into clusters. There was no significant grouping of the sources of information (results not shown), so sources were analyzed individually. Statistical analysis was performed in Stata 15 (Stata Corp., College Station, TX).

## Results

Within our target demographic, 5472 panelists who were offered the survey opened it. Of those that opened the survey 2100 started the survey and 1702 respondents completed it in entirety, for an overall response rate of 38.4% and a survey completion rate of 81.0%. We excluded 277 responses (16%) due to quality control concerns, for an overall sample size of 1425 quality responses. In Table 1 we show characteristics of our survey participants. Survey respondents reflected that of Cook County census data with 40% of participants identifying as non-Hispanic White, 24% non-Hispanic Black, 27% Hispanic and 9% non-Hispanic multi-race/other. Twenty-nine parents completed the Spanish language version of the survey. The majority of our survey respondents’ child [ren] had health insurance (91%). Of those, 53% had private and 42% had public health insurance. Across annual household incomes 26% reported < $39,999, 29% reported $40,000–$79,999, 25% reported $80,000 - $149,999, 15% reported > $150,000 and 5% did not disclose.

**Table 1:** Study demographics

	Frequency (***N*** = 1425)	Percentage (%)
Race/Ethnicity		
Non-Hispanic White	564	40
Hispanic (Any Race)	391	27
Non-Hispanic Black	338	24
Non-Hispanic Multi-race/Other	132	9
Insurance Type (March 1st)		
Private	748	53
Public	596	42
None	81	6
Income (USD)		
< $39,999	366	26
$40 k – $79,999	408	29
$80 k - $149,999	357	25
> $150 k	219	15
Did not disclose	75	5
Source of Information		
Internet	890	63
TV	853	60
Social Media	563	40
Government Agency	556	39
Family/Friends/Word of Mouth	536	38
Newspaper	367	26
Health Care Provider	337	24
Radio	319	22
Podcast	123	9
Church/Place of Worship	63	4

### Hesitancy toward a future COVID-19 vaccine

**Fig. 1:**
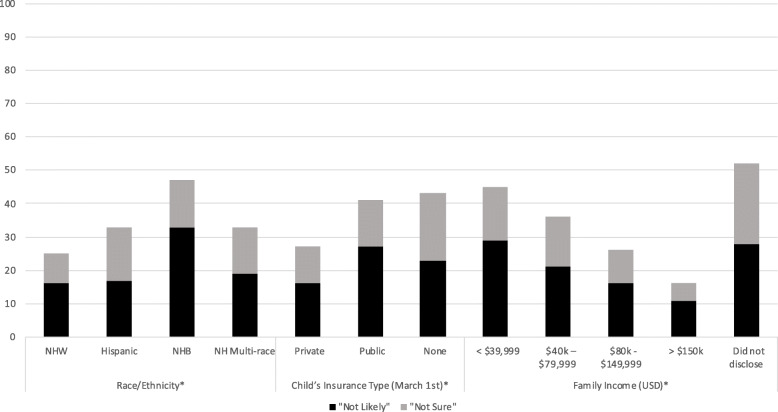
Parental COVID-19 Vaccine Hesitancy for Children in June 2020, by Demographic Characteristics. * chi-square *p* < 0.001. **Vaccine hesitancy composed of parents reporting “not likely” and “not sure” about their child receiving the COVID-19 vaccine

Overall, 33% of parents reported COVID-19 VH for their child. Figure 1 shows respondents’ hesitancy, broken down into those “not sure” and those “not likely” to receive a future COVID-19 vaccine for their child across demographic characteristics. Differences in COVID-19 VH were observed by racial/ethnic groups. Forty-eight percent of non-Hispanic Black parents, 33% of Hispanic parents, and 26% of non-Hispanic White parents were hesitant about their child receiving the COVID-19 vaccine. Of parents whose children had private health insurance 26% reported COVID-19 VH for their child. Of parents whose children had public health insurance, 41% reported COVID-19 VH for their child. Of the parents whose children had no insurance, 43% reported COVID-19 VH for their child (*p* < 0.001 for all health insurance comparisons). Parents who had an annual household income > $150,000 reported less COVID-19 VH than parents who had an annual household income of less than $40,000 (7% vs. 44% VH for children, respectively, *p* < 0.001).

### Sources of information

**Fig. 2:**
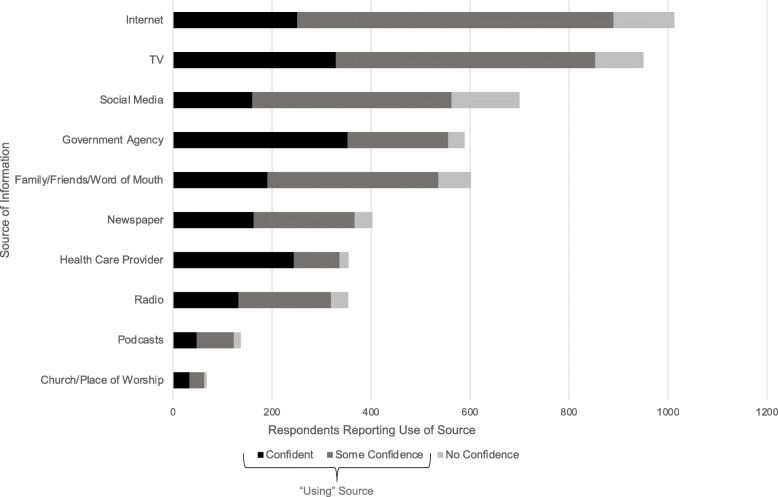
Sources of Information Regarding COVID-19 Used by Parents (*n* = 1425)

Of the 11 information source options (Fig. 2), the majority of respondents (67%) used the internet as a source of information, with only 26% of those reporting having confidence in that source of information. The greatest level of confidence was in government agencies, for which 52% of respondents reported using as a source of information and 48% of respondents having confidence in the source. This was followed by health care providers (used by 40%) with 44% of people having confidence in the source. Very few respondents selected “other”, 33 respondents (2%), and this was dropped from subsequent analyses. All information sources in which parents were confident were found to be significantly associated with lower odds of vaccine hesitancy, except for religious sources (*p* < 0.001) (Table 2).

**Table 2:** Associations of Vaccine Hesitancy for Children

	Child Vaccine Hesitancy OR (95%CI)	Child Vaccine Hesitancy aOR (95% CI)
Race/Ethnicity		
Non-Hispanic White	Reference	Reference
Hispanic	1.44* (1.08–1.91)	0.98 (0.72–1.34)
Non-Hispanic Black	2.65*** (1.99–3.53)	1.75*** (1.28–2.39)
Multiracial/Other	1.41 (0.93–2.12)	1.08 (0.70–1.67)
Income (USD)		
> $150 k	Reference	Reference
$80 k - $149,999	1.80** (1.17–2.77)	1.70** (1.09–2.64)
$40 k – $79,999	3.06*** (2.02–4.62)	2.59*** (1.68–3.99)
< $39,999	4.17*** (2.75–6.33)	2.86*** (1.80–4.53)
Did not disclose	5.70*** (3.19–10.17)	4.27*** (2.30–7.92)
Insurance Type		
Private	Reference	Reference
Public	1.93*** (1.53–2.42)	1.33* (1.01–1.75)
None	2.11** (1.32–3.38)	1.13 (0.67–1.91)
Source of Information		
Internet	0.54*** (0.43–0.68)	0.58*** (0.46–0.74)
TV	0.60*** (0.48–0.75)	–
Social Media	0.67** (0.54–0.85)	–
Government Agency	0.66*** (0.52–0.83)	–
Family/Friends/Word of Mouth	0.58*** (0.46–0.73)	0.69** (0.53–0.88)
Newspaper	0.54*** (0.41–0.71)	–
Health Care Provider	0.56*** (0.43–0.74)	0.64** (0.48–0.86)
Radio	0.62** (0.47–0.82)	–
Podcast	0.48** (0.30–0.76)	–
Religion	0.78 (0.45–1.37)	–

*p < 0.05, **p < 0.01, ***, *p* < 0.001

### Associations with vaccine hesitancy for child

Non-Hispanic Black respondents had significantly higher odds (Odds Ratio (OR) = 2.65, *p* < 0.001) of having COVID-19 VH for their child [ren] as compared to non-Hispanic white counterparts, and this relationship was maintained when adjusting for income, insurance type, and source of information (Table 2). Families with lower income had greater odds of COVID-19 VH than families in the highest income bracket. There was no interaction found between race/ethnicity and income (result not shown). Respondents with public insurance had higher odds (OR 1.9, *p* < 0.001) of being hesitant about having their child receive a future COVID-19 vaccine compared to those with private insurance – which persisted after adjusting. Finally, those who used family, internet and health care providers as information sources had lower odds of COVID-19 VH for their child (*p* < 0.001) when compared to those who did not use each source respectively. For example, parents who used family as a source of information regarding the COVID-19 pandemic had lower odds of COVID-19 VH for their child when compared to parents who did not use family as a source of information.

## Discussion

This study helps to better identify and elucidate parental COVID-19 vaccine hesitancy for children, and investigated associations between COVID-19 VH and sociodemographic characteristics including sources of COVID-19 information. Non-Hispanic Black parents, parents with lower income, and parents of children with public health insurance had higher odds of VH than comparison peers. Additionally, we found lower odds of VH in parents who used family, internet, and health care providers as sources of information about COVID-19 compared to parents who did not use these sources. Identifying which groups may be most hesitant, and which informational sources may be leveraged, are together important to disseminate accurate and persuasive information regarding a future COVID-19 vaccine to vulnerable groups. As vaccines are becoming available with emergency authorization from the FDA, and with continued spikes in COVID-19 cases, it is of the utmost importance that we are able to widely distribute the vaccine, with emphasis on groups at high, to protect all people.

In our study we found that one third of parents have COVID-19 VH for their child. Hesitancy toward a future COVID-19 vaccine is much higher than for other vaccines. Recent data shows that 6.1% of U.S. parents are hesitant about routine vaccines and 25.8% of parents are hesitant about the annual influenza vaccine [11]. There will likely be similar barriers to uptake of a COVID-19 vaccine, highlighting the importance of anticipating and responding to vaccine hesitancy during the early planning stages of COVID-19 vaccine distribution strategies [11]. A CDC strategy already underway is the “Vaccinate with Confidence” framework, which empowers healthcare professionals in reinforcing the safety and importance of vaccinations [17]. The principles of this program can be applied to routine vaccines, the annual influenza vaccine, and now the COVID-19 vaccine. In this study, we identified differences in COVID-19 VH by demographic characteristics. Vaccine hesitancy is a deadly problem, noted by the World Health Organization as a leading threat to global health in regard to known vaccine preventable illnesses [18]. While vaccine hesitancy is complex, it is imperative to explore and address specific concerns for optimal counseling and vaccine uptake [19].

Our finding of greater VH among non-Hispanic Black parents is consistent with prior research indicating that Black parents have higher hesitancy about vaccination [11, 20]. These findings are important given the disproportionate negative impact of the COVID-19 pandemic on Black communities in Chicago and throughout the United States [21, 22]. Therefore, it is of great concern that hesitancy toward a potential COVID-19 vaccine may further render Black families more vulnerable to COVID-19. Minority populations were purposefully sampled and included in COVID vaccine trials, and it is of the utmost importance to ensure the trial results are clearly communicated and the safety data is easily accessible to the public [23]. Clear communication that shares vaccine safety and efficacy data and promotes vaccination will enhance uptake of the COVID vaccine across diverse populations, as the vaccine becomes more widely available. It is crucial that further studies investigate and address reasons behind the potential hesitancy toward a COVID-19 vaccine in communities of color. Mistrust in the government and in research may be a key factor in COVID-19 VH. A study by Quinn et al. found that Black adults report hesitancy about vaccines, specifically regarding the motives of governmental agencies not “really caring about us [Black Americans]” or researchers using Black communities as “guinea pigs”, also citing the Tuskegee Syphilis experiments as a source of mistrust [24]. However, in addition to mistrust in medicine and research, other factors leading to vaccine hesitancy in Black Americans merits further study.

It is also important to note that our study showed higher vaccine hesitancy in families with lower household income, independent of race/ethnicity, which is aligned with other recent studies of vaccine hesitancy [25]. Plans for vaccine distribution and vaccine education will need to consider COVID-19 VH in groups that have been historically marginalized, to work towards equitable access to any potential COVID-19 vaccine. Further, our results should compel public health departments and community health organizations to develop effective and culturally-sensitive approaches to promote equal uptake of COVID-19 vaccine across all populations.

In addition to identifying demographic groups that may be hesitant about a COVID-19 vaccine we also characterized the association between information sources and COVID-19 VH. We found that most of the survey respondents use the internet as a source of information about COVID-19 and they reported lower COVID-19 VH. Previous randomized control trials of interventions targeting vaccine hesitancy suggested that web-based information can help improve vaccination rates, and lessen parental hesitancy towards vaccines [26, 27]. Our findings also show that using family, internet and health care providers as sources of information about COVID-19, was associated with significantly lower odds of having COVID-19 VH when compared to those not using those sources. Vaccine education interventions that use multi-modal formats (e.g., internet and healthcare providers) and are consumed by family and friends within communities may help curb COVID-19 vaccine hesitancy as we strive for herd immunity. Strategies that our participants report using are already being leveraged, including broadcasted vaccinations of public officials against COVID-19, positive vaccine messaging on social media and the internet, and public information campaigns to increase public access to scientific evidence.

Our study should be interpreted within the context of limitations, including survey validation, potential generalizability, and timing. First, although our vaccine hesitancy item was modeled after validated surveys, our survey is not validated due to the novelty of the topic. In addition, our study examines potential attitudes toward a COVID-19 vaccine in June 2020, before a safe and effective vaccine had been created and tested, or disseminated. As such, the information we have collected reflects parental intentions to vaccinate their child as of June 2020 and may not be reflective of actual decisions surrounding vaccination when their child becomes eligible for a COVID-19 vaccine. The information gained from our study can be used to inform public health initiatives to address concerns and hesitancy from parents about the COVID-19 vaccine. Future work to examine changes in parental hesitancy towards a COVID-19 vaccine over time can help to understand changing intent as COVID-19 vaccines become available to children across different age groups. Although we achieved demographic diversity in our study population, mirroring Cook County, our findings may not be generalizable to more rural areas or areas that had lower rates of COVID-19 infections. While we recognize the benefit of national samples, there are also specific benefits to representative household samples in large urban areas in which research questions can be examined with greater granular detail about the local socio-econo-environmental context for each respondent, and where samples also reflect greater demographic diversity than in typical national samples. In the case of our study, we believe that findings from our sample of households in Chicago (the 3rd largest urban area in the United States), for which we had local details about COVID case burden, are likely to be very applicable across other metropolitan areas in the nation where the burden of COVID illness and mortality has been concentrated during the pandemic. Our results may also be limited by selection bias based on the participants who chose to complete the survey. Additionally, this study occurred after protests for racial justice nationally and within Chicago and prior to the 2020 presidential election, which may have an influence COVID-19 VH, especially for non-Hispanic Black parents due to governmental mistrust [28].

However, this study’s findings regarding parental COVID-19 vaccine hesitancy for children can aid in preparation for the widespread dissemination that is necessary for a successful vaccination program. Despite this study’s limitations, this survey of parents identifies COVID-19 VH by demographic characteristics to help highlight communities that may be less amenable to receiving the COVID-19 vaccine for their child, once available. Additionally, this study synthesizes where parents receive information about the COVID-19 pandemic. The findings from this study and lessons learned from prior vaccine programs (e.g. annual influenza vaccine), including hesitancy and refusal, can help scientists, health care professionals and public health experts inform and promote a COVID-19 vaccine even before it becomes widely available in all age groups [11].

## Conclusion

COVID-19 vaccine hesitancy may limit the successful dissemination and implementation of public health strategies to mitigate the global pandemic. Since children comprise approximately one-fifth of the U.S. population, it is essential to include children and their parents in efforts to achieve herd immunity and eventually disease eradication, which are the goals of every vaccination program. The highest rates of hesitancy were found in demographic groups that have been the most adversely affected by the pandemic, and this could potentially worsen gaps in disease burden, pushing us further from health equity. Efforts to disseminate information regarding the COVID-19 vaccine should be culturally tailored and delivered through effective sources to decrease the COVID-19 illness burden in disproportionately affected groups.

## Data Availability

The datasets used and/or analyzed during the current study are available from the corresponding author on reasonable request.

## Abbreviations

CDC: Centers for Disease Control
CI: Confidence Interval
OR: Odds Ratio
VH: Vaccine Hesitancy
